# Bridging Human and Environmental Radiological Protection with the Ecosystem Services Approach: A Case Study with Clade Anthophila (Bees)

**DOI:** 10.1021/acs.est.5c16937

**Published:** 2026-08-13

**Authors:** Sarah E. Donaher, Phil Kruse, Jeffrey J. Whicker, Karine Beaugelin-Seiller, Adrienne L. M. Ethier, Cameron Jeffries, Analia Canoba, Nicole E. Martinez

**Affiliations:** † Department of Civil and Environmental Engineering, 4292 University of Tennessee, Knoxville, Tennessee 37996, United States; ‡ Department of Nuclear Engineering, University of Tennessee, Knoxville, Tennessee 37996, United States; § EDF Energy, Bristol BS32 4SQ, U.K.; ∥ Los Alamos National Laboratory, Los Alamos, New Mexico 87544, United States; ⊥ Autoritéde de Sûreté Nucléaire et de Radioprotection, PSE-ENV/SEDDER, F-13115 St-Paul-lez-Durance Cedex, France; # 6324 Canadian Nuclear Safety Commission, Ottawa, Ontario K1P 5S9, Canada; 7 South Australian Medical Imaging, Adelaide, South Australia 5000, Australia; 8 Autoridad Regulatoria Nuclear, Ciudad de Buenos Aires CP 1429, Argentina; 9 School of Civil and Environmental Engineering and Earth Sciences, Clemson University, Anderson, South Carolina 29625, United States; 10 Center for Nuclear Environmental Engineering Sciences and Radioactive Waste Management (NEESRWM), Clemson University, Clemson, South Carolina 29634, United States

**Keywords:** ecosystem services, radiological protection, radionuclides, Anthophila, bees, environmental remediation, sustainable development

## Abstract

Ecosystem services, the benefits people derive from nature, provides a conceptual structure by which to explicitly characterize and protect the coupled human–nature relationships that are central to human well-being, the integrity of ecosystems, and sustainable development. We suggest the ecosystem services concept may complement and broaden the International Commission on Radiological Protection’s currently established framework of radiological protection (RP) through intentional reflection on the interconnection between people and their environment. As operationalization of an ecosystem services approach is a notable challenge for implementation, we discuss Anthophila (bees) as a model clade for demonstrating how the ecosystem services approach may contribute to the integrated protection of people and the environment across geographies, cultures, and exposure scenarios. Assessing the impacts of radiological contamination and associated decision-making on ecosystem services has the potential to bridge human and environmental RP through adoption of a sustainable development mindset, enhanced stakeholder engagement, and improved decision-making. We provide a framework to describe how a conceptual model to combine the ecosystem services approach with RP could be helpful in supporting holistic and integrated decision-making in certain situations and demonstrate its application to a hypothetical existing exposure situation using bees as a model clade of species.

## Graphical abstract

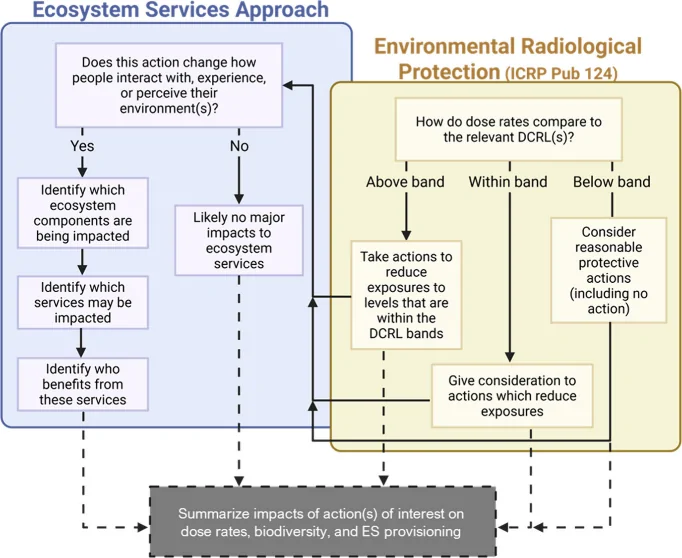

## 1 Introduction

Environmental radiological protection (ERP) is the practice of ensuring the environment is sufficiently protected from radiation and radioactive materials.^1,2^ The International Atomic Energy Agency (IAEA) explicitly includes biotic, abiotic, and cultural ecosystem components in their definition of environmental protection.^3^ Although individual governments determine their own approach and standards for ERP, the International Commission on Radiological Protection (ICRP) provides broadly accepted recommendations and takes a narrower approach to ERP, which focuses specifically on the “maintenance of biological diversity, the conservation of species, or the health and status of natural habitats, communities, and ecosystems.” ^4,5^ ICRP-developed Reference Animals and Plants (RAPs) and Derived Consideration Reference Levels (DCRLs) are internationally recognized tools for managing ERP in a manner consistent with stated goals. Other ERP procedures include the graded approach of the United States Department of Energy, which uses comparisons of radioactivity levels to biota concentration guides.^6^ We argue that current approaches to ERP can be enhanced in certain situations by the intentional consideration of the relationships between coupled human–nature systems, including the role of the environment in maintaining human well-being and the role of humanity as a beneficiary of nature. An ecosystem services approach could serve as a potential bridge between human radiological protection (RP) and ERP by providing a means by which to classify, quantify, describe, and communicate how ecological functions and processes impact humanity and, in turn, how human activities and behaviors influence ecosystems.

Ecosystem services are described in depth elsewhere^7,8^ and, for the context of this work, are simply the goods and services provided by nature from which humans derive beneficial use.^9^ Ecosystem services are typically broken into categories such as provisioning services (e.g., products from nature such as food, water, and raw materials), regulating services (e.g., processes that moderate natural phenomena such as pollination, flood control, and disease mitigation), and cultural services (e.g., nonmaterial benefits from interacting with the environment, such as recreation, artistic inspiration, and educational opportunities). A fourth category, supporting services, incorporates fundamental processes that underpin services in the other three categories, such as the water cycle, biodiversity maintenance, and primary production.

The set of recommendations provided by the ICRP with respect to the RP of the natural environment is described in, e.g., Publications 91, 108, 114, 124, and 136 and will only be briefly summarized here (Section 3).^1,5,10–12^ Although the interconnectedness of people and their environment has long been recognized, methods for characterizing and protecting coupled human–nature relationships remain challenging.^13–15^ As it is well-acknowledged that explicit consideration of the environment as both distinct from and in relation with people is beneficial for robust RP,^1,4^ adopting a graded approach to consideration of ecosystem services represents a promising opportunity to advance RP via dedicated and structured reflection on the relationships between people and their environment.^16,17^

Bees provide a useful case study to demonstrate a strategy for operationalization of an ecosystem services approach to RP. In the context of this paper, “bees” refer to the group of over 20,000 winged insect species in the clade Anthophila under the superfamily Apoidea. Other types of winged insects, such as wasps, are explicitly excluded from consideration within this paper. Located on every continent except Antarctica, bees are a convenient model clade for exploring application of the ecosystem services approach with RP.

First, bees are intricately but directly linked to a number of ecosystem services, including provisioning services (e.g., production of honey, beeswax, and bee pollen), regulating services (e.g., pollination and maintenance of biodiversity), and cultural services (e.g., apitourism, educational opportunities, and, for some individuals, an appeal related to aesthetics or intrinsic value).^18^

Second, bees are broadly used in biomonitoring to indicate the presence of contaminants such as heavy metals, pesticides, and radionuclides either through measurement of pollutant residues in honey, pollen, or tissue or through observation of some deleterious physiological responses to pollution.^19,20^ The status of bees as precharacterized biomonitors is useful for our purposes because best practices, protocols for monitoring and assessment, and previous identification of meaningful or sensitive responses are already well-documented in the literature.^20–23^

Third, much is already known about the effects of both natural and anthropogenic disturbances on the health and wellness of bee individuals and populations, including wildfires,^24^ pesticides,^25,26^ and ionizing radiation.^27,28^ External dose from gamma radiation has been linked to responses in antioxidant and immune system biomarkers in honeybees, including the concentration of relevant enzymes such as catalase, superoxide dismutase, and phenoloxidase,^29^ declines in bumblebee colony queen production,^28^ and changes in bumblebee metabolic processes critical to both individual and colony health.^27^ Bees have been suggested as a potential model organism to incorporate ecological realism into radioecological risk assessments through the use of adverse outcome pathways to predict the impact of indirect mechanisms such as trophic interactions and/or species behavior (e.g., loss of pollinating bee populations or altered individual foraging behavior of bees) on radiologically impacted ecosystems.^30^ Similarly, the available body of literature on bee ecotoxicology may facilitate a more informed series of predictions and extrapolations to describe changes in ecosystem service delivery associated with radiological contamination and associated decision-making.

Finally, bees are one of the ICRP’s 12 RAPs that are currently applied in ERP to assist in the extrapolation of exposure-to-dose and dose-to-radiation effects.^10^ Therefore, bees have well-characterized dose conversion factors and dose–effect relationships reported in the literature compared to many other wildlife species. Bees and their connection to critical ecosystem goods and services such as honey and pollination therefore serve as a helpful common point of reference between current approaches in ERP such as RAPs and DCRLs and the novel association with ecosystem services. This paper presents suggestions for the role and implementation of an ecosystem services approach with RP through the lens of bees, a well-studied and globally distributed set of species. We review the impact of disturbances on ecosystem services relevant to bees and how bee-associated ecosystem services can be readily integrated into the current System of RP and demonstrate a bee-related case study to highlight the application and operationalization of the ecosystem services approach with RP.

## 2 Relevance of Bees to Ecosystem Services

### 2.1 Regulating Services

One of the defining ecosystem services provided by bees is the regulating service of pollination, which is essential for the reproductive success of many plant species. Pollination underpins global food security and supports ecological integrity by sustaining primary productivity and promoting biodiversity through the enhancement of plant genetic diversity. A meta-analysis of animal pollinators in crop production identified 57 species contributing to the pollination of crops grown for direct human consumption, with bees accounting for 47 of these species,^31^ underscoring the predominant role of bees in agricultural systems. However, anthropogenic pressures, such as climate change, habitat degradation, and environmental pollution, compromise the delivery of bee pollination.^32^

Pesticides are a pollutant of particular concern for bee species, contributing to documented declines in wild bee populations across Europe and North America.^25,33,34^ However, other environmental contaminants such as toxic metals and radionuclides can induce sublethal or lethal toxic effects in bee individuals and populations, which may reduce their ability to provide pollinating services.^35–37^ While the effect of ionizing radiation on bee health at both the individual and population levels remains an active area of investigation that may provide future insights useful for ecosystem services assessment,^29^ current efforts may be more pragmatically directed toward understanding how remediation activities and radioactive waste management influence pollination service delivery.^38^ Integrating pollinator ecosystem services into land-use and environmental remediation planning within the context of RP offers an opportunity to reduce costs, enhance biodiversity and conservation outcomes, and safeguard public health.^39^

### 2.2 Provisioning Services

Bees provide provisioning services in the form of honey, beeswax, royal jelly, bee pollen, and other related goods. Provisioning services are often the easiest category of ecosystem services to characterize and quantify because of the relative ease of defining measurable physical metrics for these services and the ability to readily assign a monetary value to the associated ecosystem goods.^40,41^ The long-standing debate associated with the economic valuation of ecosystem services is reviewed elsewhere and will not be addressed here.^42,43^ Regardless, it is readily apparent that the presence of elevated radioactivity in an environment (either real or supposed) will influence the availability of provisioning services and any associated monetary value due to factors such as prohibited sale or use of raw materials and foodstuffs when regulatory limits are exceeded or public reluctance to use products from a region perceived as contaminated.^44–46^

Although assessment in terms of the volume, mass, or economic value of lost product is perhaps the most common method to assess the impact of ionizing radiation on provisioning services,^47,48^ we suggest that incorporating changes in the rate(s) of goods production could provide a more robust analysis. Both climatic and habitat-related changes can alter honey production at spatial scales on the order of kilometers,^49^ suggesting this may be a sensitive metric for assessing the integrity of ecosystem services in an anthropogenically disturbed environment. Exposure to elevated concentrations of metals can also alter the rates of provisioning ecosystem services derived from bees. For example, colonies treated with a 0.24 mg kg^–1^ cadmium (Cd) sugar syrup produced significantly more honey than control colonies after a 60-day treatment period, possibly because of an overall resource need reduction of the colony, as evidenced by the observed increase in dead pupae.^50^ To the best of the authors’ knowledge, no study to date has investigated the influence of ionizing radiation on the rate of honey production in a bee colony, although nectar consumption in bumblebees increased for individuals exposed to dose rates in the range of 50–200 μGy h^–1^.^27^ Further studies on the generation rates of bee-related products may therefore contribute to resolving one of the major ongoing challenges in radioecology and ERP, namely, characterizing the impact of ionizing radiation to ecological functioning and processes and other higher levels of ecological integrity.^14,15,51^

### 2.3 Cultural Services

Bees have occupied a prominent place in human art and cultural expression across the globe since antiquity.^52^ In contemporary societies, the slogan “save the bees” has become a staple of sustainability initiatives led by nongovernmental agencies, citizen science networks, and policymakers. This popularity has led to the emergence of the term “bee washing” to refer to pollinator conservation efforts lacking a sound scientific foundation.^53^ The persistent and widespread public engagement with bees, as both ecological agents and artistic symbols, highlights their unique capacity to transcend generational and cultural boundaries. While identifying universally shared values remains a challenge in environmental ethics, bees hold a widespread aesthetic appeal and broadly shared human affinity.^54^ Coupled with their near-global distribution, bees offer a useful framework for cross-cultural comparisons of both the impacts of and actions in response to radiological contamination through a shared set of cultural ecosystem services.

Environmental contamination, whether chemical or radiological, can substantially impair access to cultural ecosystem services. These impacts may include the loss of recreational or tourism opportunities, the erosion of mental health benefits derived from natural environments, and the exacerbation of pre-existing emotional stress. Moreover, such disruptions often perpetuate environmental injustices and social inequalities by disproportionately affecting marginalized populations. As previously noted, these consequences may arise regardless of whether the contamination is actual or perceived. For instance, members of the public are typically hesitant to reside or recreate in regions affected by radiological events, even following remediation efforts and expert assurances.^55^

Bees offer a diverse array of cultural ecosystem services that may be particularly well-suited for integration into radiological risk assessments and decision-making frameworks. Such services include biomimetic inspiration for architects, engineers, and musicians, the emotional and educational benefits associated with beekeeping and apitourism, and their frequent representation in artistic media such as painting, photography, sculpture, and digital art.^18,38,52^ These services are susceptible to direct disruption from environmental contamination or indirect effects stemming from decisions related to protection or remediation, such as losing access to hives during evacuation or degradation of bee habitat or food sources during environmental cleanup. One of the challenges of ERP in general and the incorporation of the ecosystem services approach in particular is identifying common approaches to facilitate guidance and implementation (i.e., operationalization). The evaluation of bee-related cultural services presents a promising, broadly relevant criterion that may be applied across diverse exposure scenarios, forms of contamination, and ecological regions.

## 3 Relevance of Bees to RP

While the ICRP’s definition of ERP focuses on biological ecosystem components, the IAEA takes a broader approach, which includes explicit consideration of nonliving ecosystem components and cultural amenities.^3^ Ecosystem services in general, and bee-related services specifically, provide a structured framework by which to simultaneously consider biotic, abiotic, and cultural ecosystem components for the purpose of ensuring sufficient protection of both humans and the environment. Furthermore, there is a growing recognition that effective human and environmental RP is crucial to support sustainable development, prompting calls for more transparent integration of sustainability principles into future ICRP guidance.^56^ Bees are a particularly relevant model clade within both current and emerging RP paradigms due to their ecological role in supporting biodiversity, their designation as one of the original ICRP RAPs, and their direct relevance to several United Nations Sustainable Development Goals (UN SDGs).^57^

### 3.1 Biodiversity

Under the ICRP definition, one of the primary goals of ERP should be to mitigate the deleterious effects of radiation such that there is “a negligible impact on the maintenance of biological diversity.”^5^ The IAEA is similarly concerned with the “conservation of non-human species, both animal and plant, and their biodiversity.”^3^ Bees are connected to biodiversity in a number of interconnected ways: they play a direct role in maintaining biodiversity in both natural and managed environments through pollination, the health of bee populations is, in turn, affected by changes in plant biodiversity in their own habitats, and the biodiversity of wild bee populations themselves can be a useful indicator of ecosystem health.^58^ These direct connections to biodiversity provide opportunities to assess bee-related ecosystem services (e.g., pollination rates, honey generation, and api-tourism) as novel end points to support the current biodiversity goals of ERP. Bee-related metrics may therefore support the transition of ERP to a more proactive and resilience-based system (e.g., declines in pollination rates indicating future plant biodiversity losses) rather than a reactive one (e.g., decline in species richness).

### 3.2 RAPs and DCRLs

ICRP Publication 108 introduced the concept of RAPs as an analogue to the established “Reference Person” framework, with the objective of providing a structured framework for understanding relationships among exposure, absorbed dose, biological effects, and ecological consequences in wildlife.^10^ A RAP is a “hypothetical entity, with the assumed basic biological characteristics of a particular type of animal or plant, as described to the generality of the taxonomic level of family, with defined anatomical, physiological, and life-history properties.”^10^ The 12 specific RAPs were selected with the intent to integrate dosimetric models with empirical data on radiation effects in animals and plants, while allowing for iterative refinement as new data was acquired. RAPs are inherently designed to evaluate environmental protection across multiple spatial and ecological contexts because reliance on a single representative species may not adequately capture variability in exposure pathways or impacts to biodiversity.

Although in practice there is significant variation in the life histories and behaviors of bee species, the ICRP Reference Bee most closely resembles a typical social bee from the Apidea family and is more generally representative of a terrestrial insect. Bees were chosen to represent terrestrial insects because they were determined to be the easiest to handle and most studied species, which similarly make them suitable for exploring the ecosystem services approach with RP.

Initial dose conversion factors in terms of μGy/day per Bq/kg were derived for each RAP for 75 radionuclides.^10^ The bee colony was included as a distinct entity in the calculation of dose conversion factors and has its own set of values separate from the individual bee. Bees had no available data at the time of initial RAP development for mortality and morbidity end points; the effect of acute gamma irradiation for beetles and wasps was substituted to predict mortality, and no data were available for the effects of irradiation on morbidity for any large insects. However, a number of studies were available to assess the impacts of radiation on reproductive success and chromosomal damage or mutations in bees and large insects. Subsequent improvements have been made since the initial development of the RAPs, including additional models for estimating exposure,^12^ advancement in the methodology for deriving RAP dose coefficients,^11^ and recommendations for radiation weighting factors for RAPs.^59^ While RAPs and ecosystem services can be addressed for separate organisms in any given ERP context, groups of species with strong ties to both concepts, such as clade Anthophila, provide opportunities for integrated assessments.

The concept of DCRLs was also introduced in ICRP Publication 108 alongside the RAP approach, with the practical application further developed in ICRP Publication 124.^5,10^ DCRLs are RAP-specific bands of dose rates within which there exists some likelihood for the occurrence of deleterious effects. We suggest that an ecosystem services approach may follow as a secondary analysis in some situations to evaluate protective actions in order to mitigate potential adverse impacts to positive human–nature coupled relationships and associated ecological functions and processes or to transparently justify decision-making in cases when higher human and ecological protection may be achieved by leaving contamination in place (for example, a contaminated but isolated and controlled site reallocated as a wildlife sanctuary and not for future public release).^39^

### 3.3 Sustainable Development

The UN SDGs are a set of 17 ambitious goals established in 2015, which address the planet’s biggest challenges such as ending poverty, promoting economic growth, and protecting the environment.^57^ The direct connections between the objectives and outcomes of RP and several of the SDGs^56^ and the role of bees in achieving sustainable development^60^ have been reviewed more thoroughly elsewhere and will only be addressed in brief here. Bees are expected to contribute to, at minimum, 15 of the UN SDGs, primarily through pollination, which supports “Zero hunger” [SDG 2] and “Life on land” [SDG 15] (UN, 2015). However, bees also contribute to additional SDGs, such as “No poverty” [SDG 1] through beekeeping as an income source, “Good Health and Well-being” [SDG 3] through the use of honey and other bee-associated goods as bioactive compounds in both traditional and modern medicine, “Industry innovation and infrastructure” [SDG 9] through bee-inspired biomimicry, and “Sustainable cities and communities” [SDG 11] from the use of bees as air-quality biomonitors.^60^

RP plays a similarly important role in achieving many of these same aims, including ending poverty [SDG 1] through the responsible protection of populations living in long-term contaminated areas, ending hunger [SDG 2] through effective remediation of contaminated cultivable land, ensuring health and well-being [SDG 3] by managing the risk of radiopharmaceuticals to the environment, promoting protection of the environment near facilities associated with the nuclear fuel cycle [SDGs 7 and 9], managing occupational and personal radon exposures [SDG 11], and protecting terrestrial ecosystems [SDG 15] through effective radiological impact assessments.^56^ The ecosystem service approach and aims associated with sustainable development can be evaluated simultaneously for many RP contexts. For example, integrated assessments of these two concepts may provide a more robust analysis of the consequences of radiological contamination or the subsequent actions taken to remediate that contamination.

## 4 Applying the Ecosystem Services Approach with ERP

### 4.1 Conceptual Framework

To facilitate the initial incorporation of the ecosystem services approach with ERP, we propose an illustrative hybrid ERP–ecosystem services framework to conceptualize the relationship among DCRLs, ecosystem services, and sustainable development. The three-pillar representation of society, economy, and environment is a widely accepted conceptual model for sustainable development (Figure 1).^61^ The ecosystem services approach is aligned with all three pillars because it addresses the components of nature that support human well-being and industries. In combination, we hope that the ecosystem services approach to ERP will support sustainable development, a stated goal of numerous international organizations such as the ICRP and IAEA.^56,62,63^

**Figure 1:**
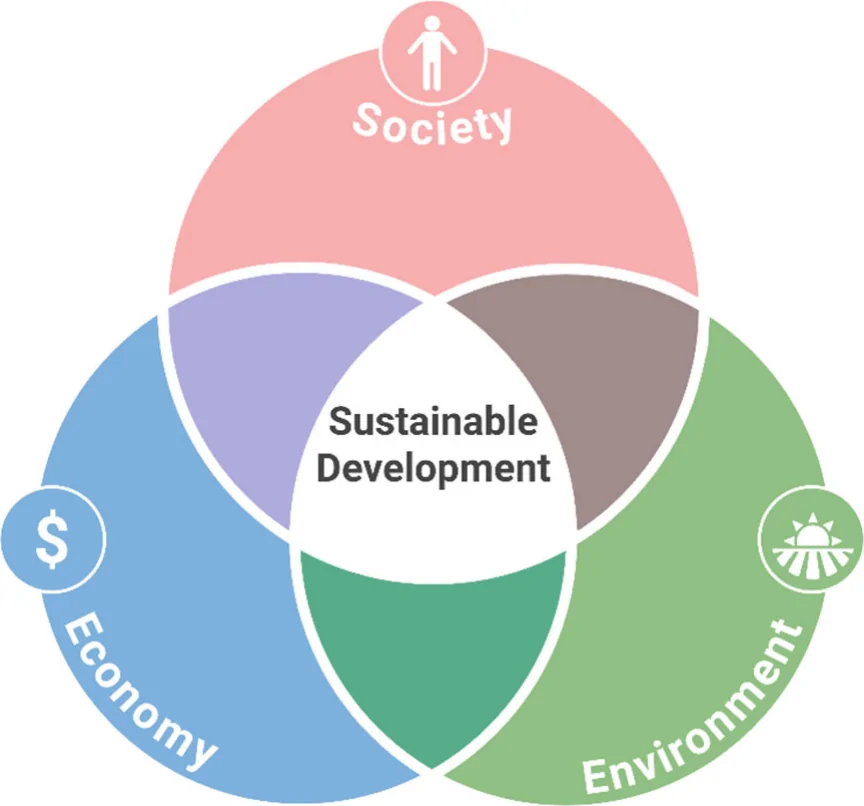
Classic three-pillar model of sustainable development (society, environment, and economy). The figure was created in BioRender by Donaher and Martinez (2026).

Figure 2 demonstrates how the ecosystem services approach can work alongside the existing tools of RP to support holistic decision making in certain situations and provide a bridge between human and environmental protection. The ecosystem services approach can be integrated sequentially with traditional human and environmental RP approaches through reflecting on the impact of proposed protective actions. Exposure situations are classified as planned, existing, and emergency (Figure 2) based on the characteristics of the situation. Although “similar procedures are used for deciding on the extent and level of protective actions, regardless of exposure situation,”^4^ the actions themselves, timelines, resources, etc. will vary, ultimately reflective of the prevailing circumstances. It is important to clarify that the application of the ecosystem services approach is not suggested as a dedicated protection goal, and it may not add value in all cases.

**Figure 2:**
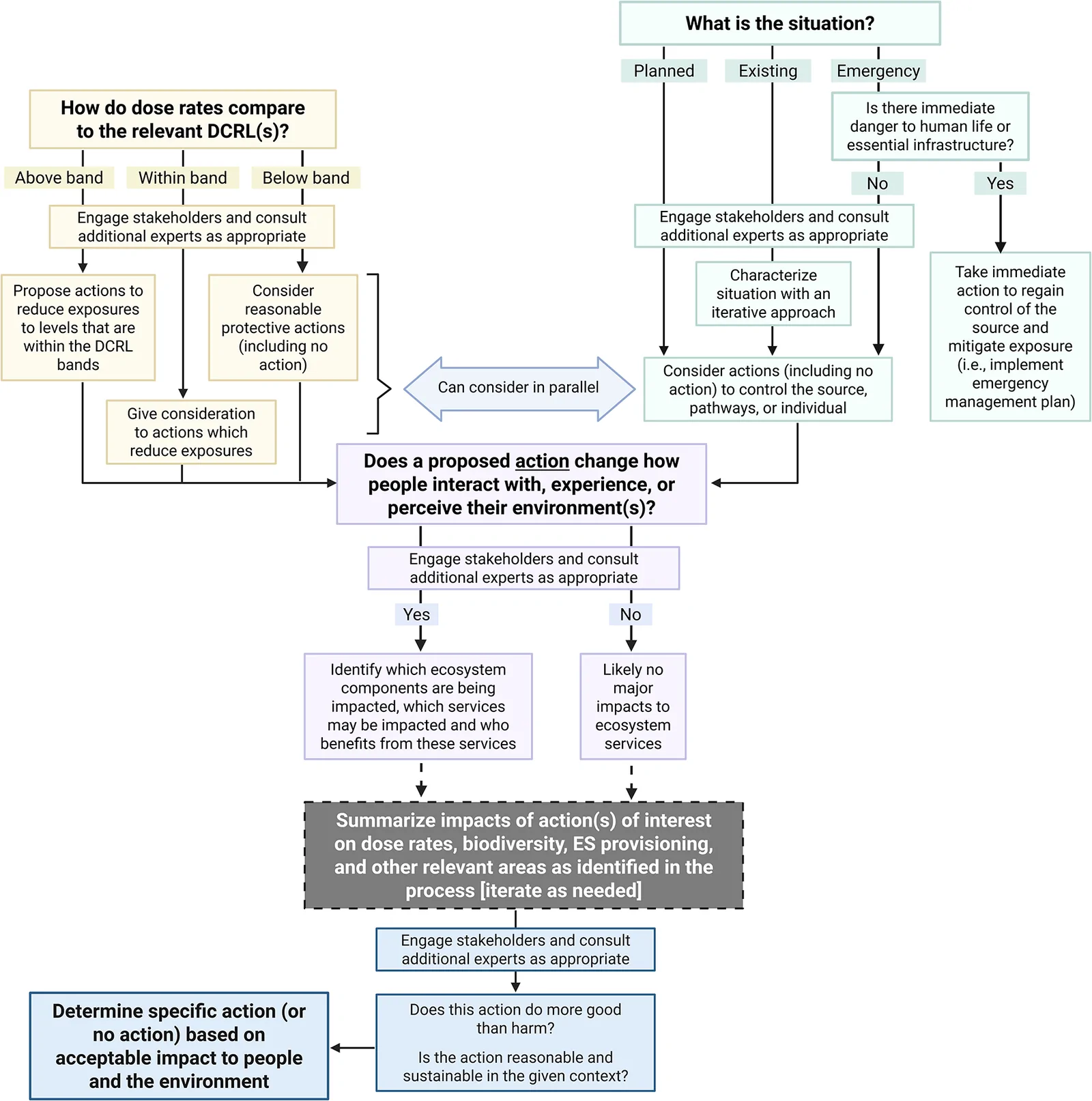
Simplified conceptual model illustrating the integration of ecosystem services into the existing approach to RP. The top right (“what is the situation?”) reflects the basic approach to managing exposure of people.^4,64,65^ The top left portion (“how do dose rates compare to DCRLs?”) reflects the basic approach to managing exposure of wildlife.^5^ Newly proposed is the explicit, dedicated consideration to how potential actions taken to reduce exposure may impact the integrity of nature or ecosystem services to inform more robust and sustainable decisions-making. Of note is that the level and extent of (1) stakeholder engagement and (2) consultation with experts from varying disciplines will vary depending on the prevailing circumstances. The figure was created in BioRender by Donaher and Martinez (2026), https://BioRender.com/eachq7z.

In traditional ERP, the initial step is to assess how dose rates compare to the relevant DCRLs for the RAPs of interest, which is more thoroughly described in ICRP Publication 124.^5^ Briefly, if dose rates are above the DCRL band, the recommendation is “to reduce exposures to levels that are within the DCRL bands for relevant populations, with full consideration to the radiological and non-radiological consequences of doing so.” If the dose rates fall within the DCRL band, “consideration should be given to reduce exposures, assuming that the costs and benefits are such that further efforts are warranted.” Finally, if dose rates are below the DCRL band, “the level of control is determined by selection of the most reasonable protection action,” which could include no action alternatives (Figure 2). The primary difference for a planned exposure situation is that “the lower boundary of the relevant DCRL band should be used as the appropriate reference point,” and for an emergency situation, it is noted that “consideration of environmental protection may not be an immediate priority” yet “consideration should be given to the environmental consequences of the possible options available to achieve adequate level of human protection,”^5^ which may include trying to preserve ecosystem services in the immediate aftermath. The ecosystem services approach may also assist in full consideration of radiological and nonradiological consequences, as relevant to dose rates above the band, and determining costs and benefits warranting further efforts, as relevant to dose rates within the band.

Regardless of the relationship between the DCRLs and the actual dose rates, some sort of action (which may include no action alternatives) may be proposed according to the recommendations outlined in Publication 124.^5^ We suggest that the next consideration should be, “Does this action change how people interact with, experience, or perceive their environment(s)?” This question is intended to inherently capture whether an action will alter ecosystem services provisioning. If the answer to this initial question is no, then we do not expect to see major impacts to ecosystem services due to the suggested action. If the answer is yes, we propose a three-step classification scheme to identify the services and stakeholders most relevant to the suggested action. First, identify which ecosystem components will be impacted by the action (e.g., human access to the environment, abiotic components, such as trees or soil, or biotic components, such as species).

Next, identify which services may be impacted by determining the major services provided by the previously identified ecosystem components. Numerous resources exist to relate ecosystem services to components, such as the United States Environmental Protection Agency (U.S. EPA)’s National Ecosystem Services Classification System (NESCS) Plus tool,^66^ the Government of Canada’s Ecosystem Services Toolkit (VNCST, 2017), and the Toolkit for Ecosystem Services Site-based Assessment.^67^ Although some frameworks for ecosystem services approaches suggest identifying impacted ecosystem services prior to identifying key ecosystem components of the relevant ecosystem services,^68^ we suggest that for RP professionals, many of whom may be newly adopting the ecosystem services approach, first identifying the key ecosystem components will assist in accurate subsequent identification of relevant ecosystem services.

Finally, identify who typically benefits from the impacted services, including any relevant stakeholders. After adopting this approach for each action of interest, the user can choose, if desired, to systematically compare the impacts of each action on dose rates and ecosystem service provisioning. Ecosystem services assessment can be tailored to the needs of a specific situation but is typically done through a biophysical, social, or economic assessment.^40^ Biophysical assessments include characterizing the structure and functions of an ecosystem as they relate to ecosystem service provisioning using quantitative monitoring and measurements such as spatial and temporal data, modeling, and mapping. Social assessments rely on the involvement of stakeholders to assess the importance of specific ecosystem services to those groups using sociological approaches such as surveys and dialogues.^67^ Economic assessments evaluate ecosystem services as a function of their monetary value using valuation methods such as market value analysis and avoided damage costs.^7^ Users should take a graded approach to any of these assessments, with the amount of invested effort being proportional to the expected risks or benefits in the context of the prevailing circumstances.^69^ For example, impacts to ecosystem services may be considered qualitatively, or the user may choose to define specific protection goals for each key ecosystem component-service combination.^68^ The assembled data should bring transparency into how decisions were made for a particular situation and serve as a communication tool to explain the benefits and consequences of each action. The ecosystem services approach described here can also be applied to actions taken to protect human health and well-being.

### 4.2 Case Study: Assessment of Bee-Related Ecosystem Services Prior to Remediation of Contaminated Soil in an Arid Environment

Applying the ecosystem services approach to remediate and restore chemically and radiologically contaminated lands necessitates the development of new frameworks and tools.^70^ Herein, we discuss a hypothetical case study to apply our hybrid ERP–ecosystem services framework to bee-related ecosystem services in a radiologically contaminated environment (e.g., an existing exposure situation). Environmental remediation and restoration generally improve the richness and abundance of wild bee populations, even when bees are not explicitly included in the planning and design phase.^71^ In turn, consideration of pollinator-provided ecosystem services has been shown to improve the outcomes of remediation and restoration of contaminated land, although notable challenges include the need to understand the specific niche of the pollinator within the ecosystem, accounting for the varying spatial scales that pollinators travel, and a lack of quantitative data on the benefits of bee-related ecosystem services to sectors other than agriculture.^38^

Our hypothetical case study considers a semiarid (total precipitation ∼ 38 cm per year) environment contaminated with a mixture of radionuclides (for example, ^3^H, ^137^Cs, ^238,239^Pu, ^90^Sr, ^241^Am, and ^tot^U) in the top 5–15 cm of topsoil, similar to the environmental and radiological conditions observed in some areas of Los Alamos National Laboratory (LANL) in New Mexico, USA.^72^ In fact, LANL maintained and monitored a collection of both onsite and offsite honeybee hives from 1979 to 1995 for the purposes of monitoring radionuclide bioaccumulation in bee tissue and honey and estimating the committed effective dose equivalent to individuals who may consume honey from offsite hives near the perimeter of the facility.^73^ In both reality and in our hypothetical scenario, shallow burial of low-level waste, initially intended to be temporary but remaining in place for decades, results in a constant flux of tritium out of the soil and facilitates the migration of other radionuclides through soil and groundwater.

Initial remediation efforts at LANL for managing some of this low-level waste included the installation of a soil cap designed to store infiltrated water until removal via evapotranspiration to limit the movement of contaminants through the environment.^74^ Over time, native vegetation, including culturally significant wildflower meadows, reestablishes on the soil cap and restores ecological functioning and services, supported by the efforts of pollinators such as bees. In reality, after removal of buried waste containers, residual soil contamination is expected and installation of these protective soil caps is a final closure action at the LANL sites, but for the sake of our hypothetical case study, we assume a follow-up performance assessment is being conducted to determine if additional actions must be taken following a decades-long waiting period postinstallation. In fact, long periods of monitored natural attenuation prior to consideration of a more aggressive remediation strategy are not unusual at, for example, legacy areas within the United States associated with Manhattan Project and Cold War era contamination or other multi-isotope sites.^74–76^

In both traditional ERP and our proposed hybrid ERP–ecosystem services approach, concentrations of radionuclides in sediment, water, and other relevant environmental compartments (e.g., vegetation) are measured to determine the dose rate(s) to the RAPs of interest (in this case, bees). This dose rate can be calculated using the ERICA Tool or other alternate approaches.^77^ Appropriate protective actions to reduce dose rates or exposure to other hazardous, nonradiological contaminants, if desired, can then be considered. If the installation of the soil cap in our hypothetical system is not performing to desired levels, one potential protective action may be the excavation of the top 5–15 cm of soil to remove the areas of highest radiological and chemical contamination, which would include removal of the onsite, but not offsite, managed hives. It is important to note that this action may be appropriate even if dose rates fall below the DCRL, if it is reasonably desired to further reduce dose rates through additional protective actions for reasons such as stakeholder concerns or regulatory considerations (Figure 2). The ecosystem services approach can then be applied to determine the relative impacts of soil excavation versus no action alternatives to ecological and human well-being by identifying impacted ecosystem components and services beneficiaries. This assessment may be primarily biophysical, wherein bee-related ecosystem services (e.g., rates of honey production; perceived usefulness of bees as a biomonitor; pollination of crops or wildflowers) are assessed in conjunction with traditional biodiversity and abundance metrics (e.g., species abundance and distribution; contaminant residues in tissues and pollens); social, wherein stakeholders such as apiarists, farmers, educators and learners, consumers, governmental agencies, and researchers are engaged through formal or informal measures; or economic, wherein the monetary losses and gains associated with remediation, changes in ecosystem services, but other relevant factors are considered.

Assessments will be highly site-specific and may incorporate unique cultural or geographic features. In our hypothetical case study, the ecosystem components of interest are the bee hives and wildflower species that serve as their food source. It is apparent that other ecosystem components that provide ecosystem services will also be impacted by the proposed remediation strategy (e.g., soil and its associated services such as carbon sequestration, nutrient cycling, and waste recycling), but we have chosen to focus on bee-related ecosystem component–service pairings (Table 1). In our hypothetical example, the wildflower meadows provide a culturally and economically valuable habitat by providing a uniquely complex and biodiverse habitat within the broader semiarid environment and potentially bringing in tourism. The inhabiting wildflower meadows would be negatively impacted by the proposed remediation strategy both directly due to significant physical disturbance during excavation and indirectly by the removal of onsite hives and associated bee pollination after the excavation. However, the effects on the associated wildflower ecosystem services are neutral or mixed. Although the removal of the wildflowers would eliminate a significant food source for bees, the service of honey and other goods production is not impacted because harvesting is not permitted from these locations. Whether harvesting is not permitted due to radiological concerns or site access is a relevant site-specific consideration. Although removal of the wildflowers and pollinating species has a hypothetical negative impact on the cultural services of inspiration from nature and improved mental well-being, there is also the potential for improved mental well-being to members of the public via the removal of perceived onsite radiological contamination. Assessment of cultural services would be especially important for sites such as the one for which this hypothetical case study is based on because it borders an area sacred to the San Illdefonso Pueblo, which would likely be impacted by onsite remediation.

**Table 1:** Illustrative Matrix of the Affected Ecosystem Components, Services, and Beneficiaries for the Described Hypothetical Remediation Case Study

affected ecosystem components	affected ecosystem services	outcomes of alternative #1: removal of residual radioactivity via soil excavation	outcomes of alternative #2: no action	affected beneficiaries
abiotic components: wildflowers and other bee food sources	honey and other goods production	neutral impact; honey and other bee-produced goods are not available for public consumption from this contaminated area		scientists and other researchers, consumers, apiarists, farmers
	inspiration and mental well-being from nature	mixed impacts; removal of hives causes loss of bee-related cultural services such as wildflower blooms but removal of the residual radioactivity may promote mental well-being of public		members of the public
biotic components: bee hives	pollination of crops and wildflowers	highly negative impact due to the removal of hives, particularly for wildflower pollination	highly positive impact due to the preservation of hives; no evidence of population-level impacts to wildflower pollination at the current dose rates	scientists and other researchers, consumers, apiarists, farmers, members of the public
	availability of bee biomonitors	highly negative impact due to the removal of hives	highly positive impact due to the preservation of hives	scientists and other researchers
site access	inspiration and mental well-being from nature	mixed impact; removal of the residual radioactivity may allow for increased site access but loss of hives reduces wildflower coverage, thereby reducing the value of site access	negative impact; site access remains restricted	members of the public

The proposed remediation strategy would also necessitate the removal of the onsite bee hives, thereby impeding services related to pollination and the availability of bees to serve as biomonitors, whereas these services would be preserved under the no-outcome scenario (Table 1). Finally, if removal of the residual radioactivity permits site release, either restricted or unrestricted, then the delivery of cultural ecosystem services associated with being in the presence of nature, as previously described, could be either negative or positive depending on the individual stakeholder (Table 1). Ultimately, it is critical to consider each situation on a case-by-case basis. From the perspective of the agricultural sector, an individual bee hive has limited value, as farmers are able to purchase bee boxes cheaply and efficiently. However, in a semiarid or arid environment as described here, members of the public, who represent key stakeholders in ERP, viewed the establishment of bee hives and wildflower meadows as an added value to their environment, which would not be easily replaced following intensive remediation. An ecosystem services assessment ultimately facilitates discussion around topics that may not otherwise be considered such as the likelihood for postexcavation recolonization by native plants or bees, stakeholder perceptions of radiological hazards, or mitigation options such as codesigning remediation boundaries with Indigenous groups or timing excavation outside of flowering seasons. There are also considerations between native vs disturbed land (e.g., while a bee may not distinguish between flowers in early versus late ecological succession, native land tends to provide more robust ecosystem services than disturbed lands and may possess a greater intrinsic or anthropogenically assigned value due to its undisturbed status) and natural vs managed environments (e.g., parks and farmland are already under the influence of direct anthropogenic drivers, and therefore it may be more justifiable to enact aggressive remediation strategies in these areas compared to wild or semiwild habitats).

Culturally and ecologically important species have long been monitored as bioindicators in radiologically impacted areas, but there is a notable lack of commonality, consistency, and structure in these types of assessments over space and time.^78^ Most critically, the impacts to ecosystem services (as opposed to direct monitoring of bioindicators) are rarely considered in these types of assessments and represent a crucial area of interest for advancing future RP. A significant challenge in adopting an ecosystem service framework in RP is resolving some of the uncertainties associated with the approach. There exists conceptual and definitional uncertainty inherent to the ecosystem services approach associated with ambiguity in definitions and frameworks, subjectivity of values because many services depend on human perceptions (especially cultural services), and unclear linkages to radiological end points.^17,43^ A lack of direct metrics between services and universally accepted indicators, variability in proxy measurements, and scaling issues also introduces measurement uncertainty.^38,40^ Despite these challenges, assessing ecosystem service provisioning directly may be a more effective and accurate end point in ecological risk assessments and restoration monitoring than traditional biodiversity metrics due to the complexity associated with linking environmental change, biodiversity, and human well-being.^70^

## 5 Future Considerations

The monitoring and assessment of ecosystem services provide an additional tool to aid holistic and integrated decision-making associated with RP. Drawbacks to the ecosystem services approach include an inability to fully account for the intrinsic value of nature and notable challenges associated with monetary valuation,^17,43^ but a graded or proportional approach to ecosystem services assessment remains a useful tool for engaging stakeholders and supporting decision-making in some situations, particularly for cultural services that can be highly site-specific. Bees provide a globally shared set of regulating, provisioning, and cultural ecosystem services through which to analyze this implementation within the existing standards of RP. The roles that bees play in both human societies and ecological hierarchies are numerous, and their multifaceted characteristics provide opportunities for flexibility when determining how to incorporate them with RP and environmental dose assessments.

In this paper, we primarily consider bees in their role of charismatic wildlife, or species that tend to elicit positive emotional responses from people. However, some species of bees may be pest, nuisance, or invasive species that cause real or perceived harm to the environment or people. For example, American and Australian bee species may be threatened by the presence of invasive European honeybees (*A. mellifera*), which, although valued for their honey production, represent a source of direct competition for native species.^79,80^ Some species may also cause negative impacts to human mental and physical well-being. For example, individuals in subfamily Xylocopinae within the genus *Xylocopa*, colloquially known as carpenter bees due to their nesting behavior within wood and other hard plant material, may cause damage to built structures such as decks or fences. Such “ecosystem disservices,” the harm or negative impacts that nature may cause to people, are not as frequently considered in decision-making as their positive counterparts^81^ but may be a relevant consideration for some radioecological assessments or decision-making.

Additionally, while we have discussed bees as wildlife species, they may also be kept as livestock or pets, both of which are relevant for the System of RP and likely to be an area of interest in future recommendations and guidelines.^82^ ICRP Publication 153 suggests that, based on the ethical values central to the System, pets and livestock kept under human stewardship are owed the accountability of their human caretakers to be responsibly used, managed, and protected. Therefore, there may be instances when it is appropriate to protect bees in a manner more consistent with livestock and/or pets than wildlife.^83^ However, on the basis of current guidelines, it is not clear when, how, or by whom that distinction should be made. In addition, if and what distinction should be made between the protection of natural and managed (e.g., grazing lands and urban parks) ecosystems, if any, is unclear under the current ERP standard practice.

Insects in general are typically quite beneficial for human societies yet are frequently demonized despite their numerous benefits. Similarly, ionizing radiation, even when put to peaceful purposes such as nuclear energy and medicine, often holds a negative perception among the public. It therefore seems apt that we may make a connection between bee-related ecosystem services and RP to advance sustainable development and generally contribute to human mental and physical well-being. As with sustainable development, operationalizing the ecosystem services approach is logistically and practically challenging and requires significant initial investment to ensure meaningful application and added value. We hope the guidance contained within this paper will encourage adoption of the ecosystem services approach in connection with RP to promote transparent decision-making and improve communication and educational opportunities for stakeholders by demonstrating how bees can provide a common perspective through a globally shared set of ecosystem services.
